# Pulse width modulation-based TMS: Primary Motor Cortex Responses compared to Conventional Monophasic Stimuli

**DOI:** 10.1016/j.brs.2022.06.013

**Published:** 2022-07-02

**Authors:** Majid Memarian Sorkhabi, Karen Wendt, Jacinta O’Shea, Timothy Denison

**Affiliations:** 1 MRC Brain Network Dynamics Unit, Nuffield Department of Clinical Neurosciences, University of Oxford, Oxford, OX1 3TH, UK; 2 Wellcome Centre for Integrative Neuroimaging (WIN), Oxford Centre for Human Brain Activity (OHBA), University of Oxford Department of Psychiatry, Warneford Hospital, Warneford Lane, Oxford, UK; 3 Department of Engineering Science, University of Oxford, Oxford, OX1 3PJ, UK

## Introduction

Transcranial magnetic stimulation (TMS) is a non-invasive method of stimulating and modulating the nervous system. Most TMS devices are limited to predefined pulse shapes, such as monophasic or biphasic cosine-shaped pulses. Recently, the use of state-of-the-art power electronic instruments has permitted more control over the waveform parameters in several newer devices [1] [2] (for a review of recent advances see supplementary file). A technique using pulse width modulation (PWM), called programmable TMS or pTMS, enables the approximation of a wide range of arbitrary pulses [3] [4]. However, PWM-based pulses will introduce higher frequency harmonics, but our hypothesis is that cell dynamics will attenuate them.

The goal of this study is to use PWM to approximate conventional single-pulse monophasic TMS and assess the comparability of effects of both pulse forms on motor evoked potentials. The conventional monophasic pulse of a Magstim 200^2^ stimulator was approximated by a custom-built PWM-based TMS device (pTMS2), making use of the modular device topology described in [4]. Computational modeling, resting motor thresholds (RMT), motor evoked potential (MEP) amplitude, latency and input-output (IO) curve measurements were compared within the same participants for both devices.

## Materials and Methods

### TMS devices

**Figure 1:**
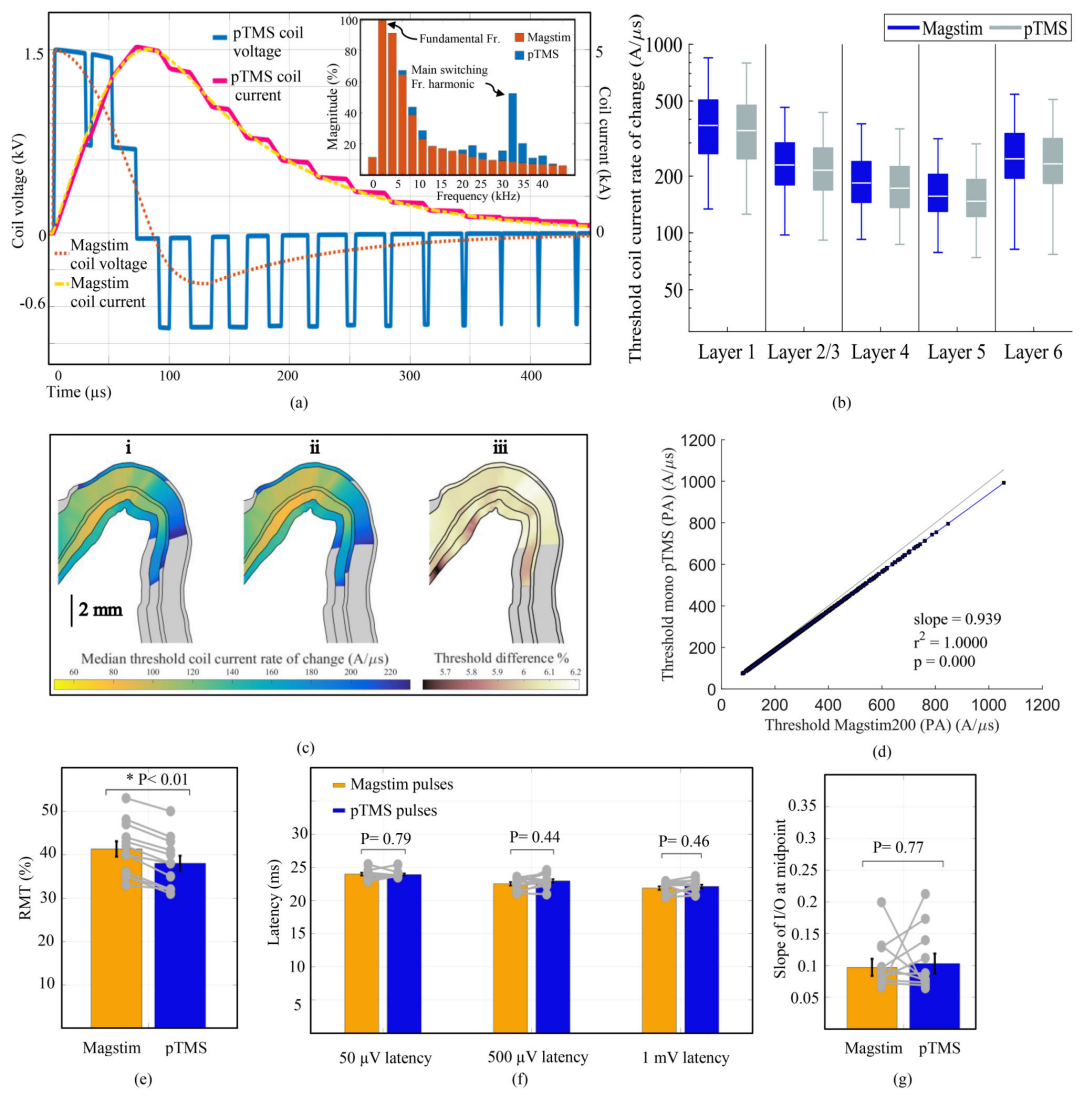
Comparison of the Magstim 200^2^ and pTMS2 system outputs and modeled responses for monophasic stimuli. (a) PWM coil voltage and current waveforms generated in the pTMS architecture in comparison with the Magstim waveforms. To approximate the monophasic pulse, the pTMS2 device synthesizes a staircase waveform using the PWM method. The voltage produced is a multi-step approximation of the target waveform. The frequency spectra of the Magstim and pTMS voltage waveform are also shown here. As expected, a PWM-equivalent pulse will have additional harmonic components. The largest harmonic is observed at 32 kHz (For more detail, see supplementary file) and is about 50% of the fundamental harmonic, while the high-frequency component corresponding to the Magstim pulse at the same frequency is 10%. The main switching frequency is 13 times higher than the main pulse frequency, and the dynamics of the neurons will attenuate them. It is important to note that the monophasic stimuli generated in conventional TMS devices, which are damped sinusoidal pulses, are not pure sine pulses and have high-frequency components. The y-axis represents a percentage of the fundamental frequency. (b) The modeled neural activation thresholds within the cortical area representing the hand muscle are shown in log scale, where blue represents the data from the Magstim 200^2^ and grey from the pTMS2. Each boxplot includes the data from five clones within each layer with the outliers removed. (c) The median thresholds of the change in coil current for the six cortical layers are shown on a 2D cross-section of the crown of the pre-central gyrus for the pulse waveforms from (i) the Magstim 200^2^ stimulator and (ii) the pTMS2 device. (iii) shows the modeled percent difference in median thresholds between the Magstim and pTMS pulses. (d) Correlation between the threshold coil current rate of change for the two pulses, with the linear regression displayed in blue and a line with a slope of 1 in grey for comparison. (e) Measured resting motor thresholds of the conventional monophasic pulse and its modulation equivalent generated by the pTMS device. To calibrate the MSO of the two devices, the positive peak coil voltage of the pTMS device was compared with the Magstim device. (f) Average MEP latencies were calculated for 50 μV, 500 μV and 1 mV peak-to-peak MEP amplitudes. While the MEP latencies varied with amplitude, they did not differ between the devices at any amplitude. (g) The slopes at the midpoints of the IO curves are shown for both pulse types. MEP measurements below 20 μV were set to 20 μV, as this was the lowest amplitude that was distinguishable from EMG signal noise. The MEP measurement was repeated 15 times for each amplitude, and the order of devices for the IO curves was counterbalanced to avoid order effects. For (e), (f) and (g), bars and whiskers show mean and standard error, respectively, with individual data points overlaid in grey. * indicates statistical significance.

We used a pTMS2 device that cascades two of the inverter cells introduced in [3] and generates magnetic pulses with five voltage levels, with no output filter. The conventional monophasic pulses were generated with a commercial Magstim 200^2^ (Magstim Co., UK). Both devices were connected to the same 70 mm figure-of-eight coil (Magstim Co., P/N 9925–00). The simulated output pulses of the two devices are shown in Figure 1a (and Figure S4).

## Physiological Response Models

To understand how the PWM stimuli interact with neural tissues, a computational model was applied before conducting the in-human study. This model also determined how many voltage levels are sufficient to approximate conventional TMS pulses with PWM-based pulses. The model, which integrates morphological neural models with transcranially induced electric fields [5], is used to compare the neural response to the Magstim and pTMS pulses, similar to a previous study [6] (see supplementary file for more details). The temporal waveforms were simulated using MATLAB Simulink models adjusted to replicate the stimulation pulses of the devices used in the in-human study. The activation thresholds for PWM and conventional TMS pulses were calculated.

## In-human study

### Participants

The pTMS device and testing procedure were approved by the Central University Research Ethics Committee (CUREC), University of Oxford (R75180/RE002) to conduct this in-human study. Twelve healthy participants (mean age: 28.6 years, range: 22–37 years; 4 male) gave their informed consent to participate in the study. All participants were right-handed as assessed by the Edinburgh Handedness Inventory (Oldfield, 1971), had no current significant medical condition, and reported no other contraindications to TMS.

### Procedure

Within each session, conventional and PWM stimuli were applied using the Magstim stimulator and pTMS devices, respectively, counterbalancing the order. The participants were seated in a chair with their arms resting on a pillow on top of a table in front of them. The coil was held by the operator, positioned over the left primary motor cortex and oriented at 45° to the midline with the handle pointing posteriorly. At the beginning of each session, the motor hotspot was determined with the Magstim stimulator, defined as the optimal scalp position where MEPs could be elicited in the right first dorsal interosseous (FDI) muscle. A Brainsight neuronavigation system (Rogue Research Inc., Montreal, Canada) was used to track the position and orientation of the coil.

Electromyography (EMG) was recorded from the FDI of the right hand in a belly-tendon montage (for amplifier and filter details, see supplementary file). To find the motor-hotspot, the pulse timing and delivery was controlled by the experimenter using the foot pedal. Then, to confirm the hotspot, and to also find the RMT and the I-O curve, the pulses were applied automatically via scripts in Signal version 7.01 (Magstim device) and Control desk (pTMS2 device) software at varying inter-pulse intervals between 4.25 to 6 seconds.

To determine the RMT, 10 pulses were applied at each intensity and the EMG traces were inspected visually in real time. The stimulator outputs were adjusted manually to determine the minimum intensity required to evoke an MEP of ≥50 μV peak-to-peak amplitude in 5 out of 10 consecutive trials.

For the IO curve, 15 MEPs at each intensity up to the maximum voltage achievable by the pTMS2 device were measured. Similar to other recent work [7], TMS stimuli were applied in increasing order from low to high intensities in steps of 3% of the maximum stimulator output (MSO) of the Magstim 200. This fixed order of stimulation was chosen to compare the devices because the intensity of the pTMS2 device needs to be adjusted manually. The coil was lifted from the participants' heads during the 1-minute rest time between each set of stimuli.

### Data analysis

For statistical analysis, we used repeated-measures ANOVA. The variables analyzed were: RMTs; input-output curves; MEP latencies with peak-to-peak amplitudes of 50 μV, 500 μV and 1 mV, defined as the time point where rectified EMG signals surpass a mean plus two standard deviations of the 100 ms pre-stimulus EMG level [8]. The data were log-transformed, similar to [8], and a Gaussian-type least-squares curve regression with four parameters was utilized to fit the data points of each participant individually (see supplementary file).

## Results and discussion

### Physiological response models

Figure 1(b) displays the thresholds for the neuron models in each layer within the cortical hand muscle representation as boxplots. The median excitation thresholds for both waveforms across a 2D cross-section of the pre-central crown is shown in Figure 1(c). The models predict the activation thresholds to be 5.6-6.2% lower for the pTMS pulse than for the Magstim pulse. Linear regression between the thresholds for the two pulse types revealed a strong correlation (r^2^= 1.000, p= 0.000) with a slope of 0.939 (Figure 1(d)), indicating a consistently lower threshold for the pTMS pulses.

### MEP measurements

The RMTs, expressed as a percentage of the equivalent Magstim maximum stimulator output, were 41.34 ± 6.07% (mean ± standard deviation) for the Magstim pulse and 38.00 ± 5.91% for pTMS stimuli (Figure 1(e)). The pulse shape had a significant influence on the RMT (F_1.11_= 115, p< 0.01). Notably, for all participants, the RMT for PWM pulses was (approximately 3%) lower than for the sinusoidal monophasic Magstim device pulses, a trend suggested by the modeling results. Using Cohen's d effect size, the difference between the RMTs was determined as a medium effect (d= 0.53). The difference in RMT may be related to the sharp edges and higher amplitude in the negative phase of the PWM pulses; another study has reported similar results for rectangular pulses [9].

The MEP latencies did not differ between devices; for 50 μV MEPs: F_1.11_= 0.07, p= 0.79, for 500 μV: F_1.11_= 0.65, p= 0.44, and for 1 mV: F_1.11_= 0.58, p= 0.46, as shown in Figure 1(f).

### IO curves

It has previously been reported that the stimulus shape affects the slope of the IO curve at the midpoint [8] [7]. Across the participants, the slopes of the IO curves did not differ between the devices (F_1.9_= 0.08, p= 0.77), as displayed in Figure 1(g).

### Side effects

No adverse events occurred during or after the stimulations. After the experiment participants were asked to verbally report any differences they noticed between stimulation with the two devices. Participants did not report any subjective difference in experience, apart from a difference in the sound during pulse firing that was reported by all participants, which is most likely due to high frequency harmonics in PWM devices.

## Limitations

PWM TMS pulses promise more flexibility in pulse generation but require further evaluation. The pTMS2 device is currently limited to lower stimulation amplitudes than the Magstim 200^2^ for the pulse width used here, which precluded data collection from individuals with very high thresholds. The maximum pulse amplitude of pTMS2 was 1600 V, compared to the maximum outputs for the Magstim Rapid, MagVenture MagPro, and Magstim 200^2^ which are approximately 1650, 1800 and 2800 V, respectively [9]. As the intensity of the pTMS2 device is adjusted manually, it is difficult to randomise stimulation intensities which may lead to hysteresis effects. To minimise this risk, stimuli were delivered in random intervals and participants had at least one-minute rest between each set of stimuli [10]. Additionally, measurements of the auditory signature are necessary for a future comparison of residual artifacts.

In summary, the measured motor responses suggest no difference in MEP latencies or IO curve slopes, but the RMT for PWM stimuli was approximately 3% lower across participants compared to the RMT for the Magstim device.

## Supplementary Material

supplementary

## Data Availability

The data and relevant scripts are available on the Open Science Framework (https://osf.io/5ry92/).

## Highlights

- The PWM method enables the digital synthesis of TMS patterns and shapes
- Suprathreshold PWM magnetic pulses were well tolerated by the participants without adverse events
- RMTs and MEPs were compared for PWM and conventional stimuli
- Thresholds were 3% lower with PWM pulses than with conventional pulses
