# Supplementary material for: Pulse width modulation-based TMS: Primary Motor Cortex Responses compared to Conventional Monophasic Stimuli
Source: Brain Stimul. Author manuscript; Available in PMC 2024 Dec 5. (PMC7617001; doi:10.1016/j.brs.2022.06.013)
Supplement: supplementary [file EMS177376-supplement-supplementary.pdf]

SUPPLEMENTARY FILE

<u>Literature review of recent TMS technology</u>

Control over the temporal aspect of magnetic stimuli can increase the neuromodulation efficacy and provide selective targeting of neuronal populations and their activation characteristics. flexTMS and related approaches, such as cTMS [1] [2], use bridge configurations of transistors, typically insulated-gate bipolar transistors (IGBT), to generate nearly rectangular shapes and to achieve control over the pulse shape. However, these machines are still limited in their generation of pulse waveforms and cannot approximate conventional sinusoidal stimuli, to perform already approved clinical protocols. Voltage decay at the beginning and end of each pulse for the flexTMS, and transfer charge from one capacitor to the other for the cTMS, would challenge the systems' performance [3]. A bridge-based TMS device is also used in multi-channel TMS systems for electronically shifting the electric field (E-field) [4] [5] [6], or for a closed-loop TMS–EEG set-up [7]. These systems focused on generating near-rectangular and single-pulse protocols.

A modular multilevel inverter (MML) topology was suggested for the TMS devices to generate near-rectangular and staircase-approximated magnetic stimuli cascading H-bridge modules and a common DC source to charge all pulse capacitors [8] [9] [10]. The bridges would be charged one at a time by bypassing the remaining bridges. Additionally, the pulse capacitors' voltage balance and the circulating current in MML structures must be carefully addressed which may require additional circuit components, and sophisticated control algorithms [11]. Therefore, using a common capacitor charger may limit TMS system dynamics for high-frequency repetitive TMS protocols.

The presented pTMS2 device uses two cascaded H-bridge (CHB) inverters with two galvanically-isolated chargers. Also, using the pulse width modulation (PWM) concept in this device enables the imitation of any arbitrary pulse waveform. Isolated DC sources can charge

the bridges at the same time without a voltage balance issue. Therefore, a CHB-based TMS device performs better in high-frequency TMS protocols, by faster and direct charging of the pulse capacitors. The proposed CHB-based TMS device and the PWM method are viable solutions for multi-channel TMS systems that can both change the effective pulse intensity for shifting the E-field locus and generate a burst of various pulses [12].

For the pTMS2 device used in this study, the multicarrier phase-shifted PWM (PS-PWM) method is used to create the switching pulses for the IGBTs. For the N=2 cascaded H-bridges and the triangular carrier frequency of F_{PWM}=8 kHz, the main switching harmonic in the output voltage will be located at 2*N* F_{PWM} =32 kHz, as evident in Fig.1a. For more detail, see [13] [14].

Different modeling and in-vivo experiments have been performed to investigate the behavior of neurons exposed to high-frequency electric fields. A single-compartment computational model of a neuron, such as the Hodgkin and Huxley model (with parameters adapted to mammalian neurons) shows the neural membrane is more sensitive to low-frequency components relative to high-frequency ones and the neurons show a weak tendency of firing for the high-frequency stimuli [15] [16]. In-vivo transcranial stimulations have also indicated that the membrane potential of neurons undergoing high-frequency stimulation did not change considerably compared to the baseline membrane potential [17] [18]. Therefore, for the PWM-based TMS pulses, as the main switching frequency is 13 times higher than the main pulse frequency, the dynamics of the neurons will likely attenuate the high-frequencies. The remaining high-frequency components are a possible cause of the observed difference between the RMTs.

Physiological response model

The model used in this study is described in detail in [19] and is available on Github [20]. In brief, it relies on the assumption that the quasi-static approximation holds for neural stimulation, allowing the separation of the spatial and temporal components of the induced electric field. First, the spatial component of the electric field of a Magstim figure-8 coil is calculated using SimNIBS. Morphological models of neurons are then placed into the region of interest and the quasi-potentials at the model compartment centers calculated and applied to the neuron models as extracellular potentials in the NEURON v7.4 simulation software [21]. For the temporal component, the temporal waveforms are simulated in MATLAB Simulink using the stimulator circuits to replicate the stimulation pulses used in the in-human study. The extracellular potentials are then scaled by the temporal waveforms and used to calculate the membrane potential of each neuron compartment. A binary search algorithm is used to scale the coil current's rate of change at the pulse onset to find the activation thresholds of the neurons (defined as the membrane potential of at least three of its compartments crossing 0 mV with a positive slope) for PWM and conventional TMS pulses. Recording the TMS system outputs and finding the threshold values with the actual waveform, instead of simulation, could minimize the computational error, as the modeling of transient switching characteristics under inductive load conditions, such as spikes and ringing effects, would not be straightforward. However, simulated waveforms were used here as the modeling was also performed to determine how many voltage levels are sufficient to approximate conventional TMS pulses with PWM-based pulses, before prototyping the pTMS device.

Further details of the model can be found in [19] and further details of this implementation in [22].

Electromyography

Electromyography (EMG) was recorded from the FDI of the right hand by positioning disposable neonatal ECG electrodes in a belly-tendon montage, with the ground electrode over the ulnar styloid process. The EMG signals were recorded using a D440 Isolated Amplifier (Digitimer, Welwyn Garden City, UK), a Micro1401 (Cambridge Electronic Design, Cambridge, UK), a Digitimer HumBug Noise Eliminator, which attenuates the unwanted 50 Hz line noise by subtracting a replica of the measured noise from the input signal without filtering, and Signal version 7.01 (Cambridge Electronic Design), with a 16-bit resolution at a 10 kHz sampling rate, an amplifier gain of 1000 and a 10-1000 Hz filter, as shown in Fig S1.

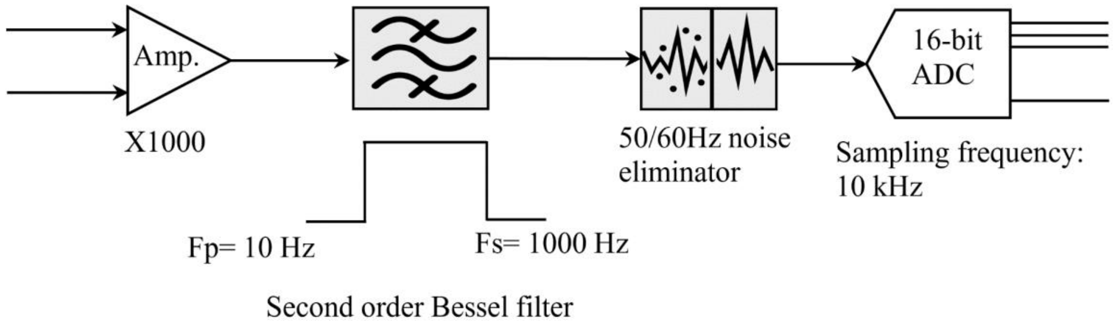

Figure S1 The EMG recording block diagram

The filter used in the Digitimer D440 device is a second-order Bessel filter. Fig S2 shows the poles and zeros of the filter. Except for the filter in the Digitimer D440, we did not apply any other filter to the measured EMG signals. Example recordings for one participant are shown in Fig S3.

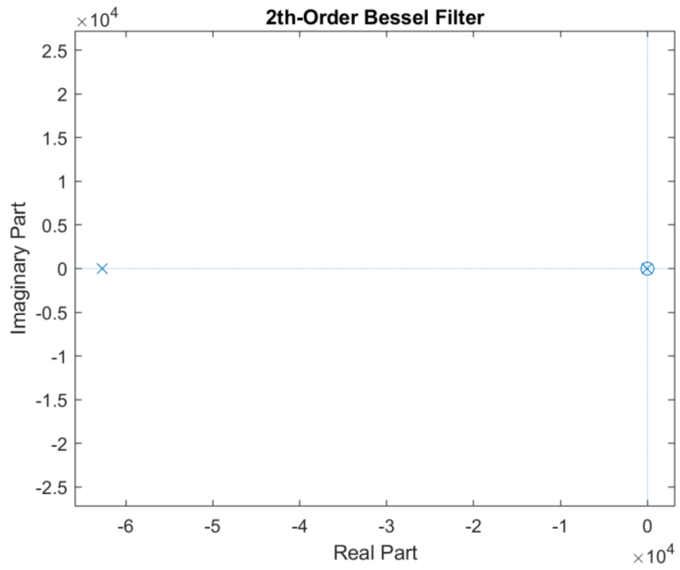

Figure S2 The poles and zeros of the utilized second-order Bessel filter

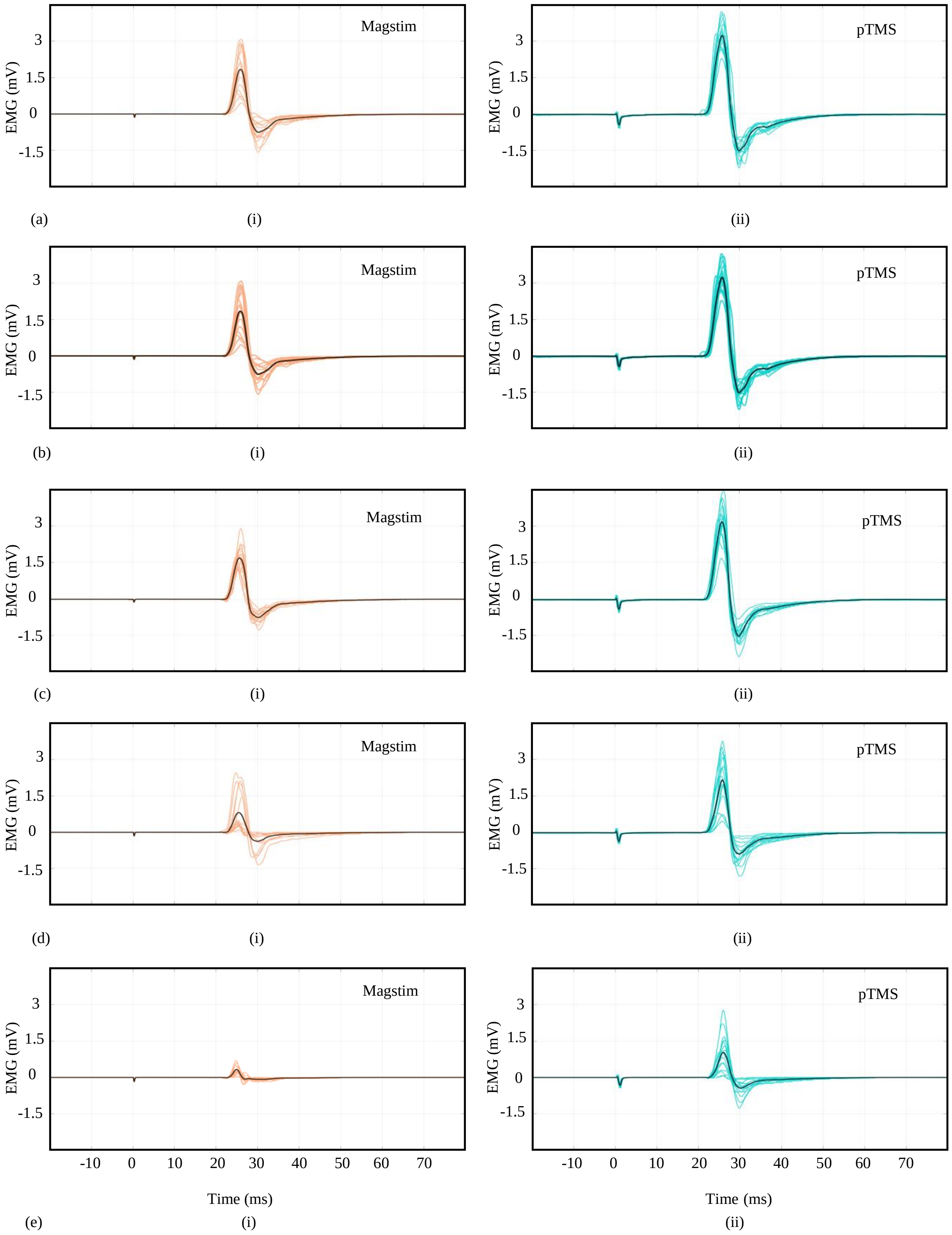
Magstim
pTMS
EMG (mV)
3
1.5
0
-1.5
-10
0
10
20
30
40
50
60
70
Time (ms)
(a)
(b)
(c)
(d)
(e)
(i)
(ii)

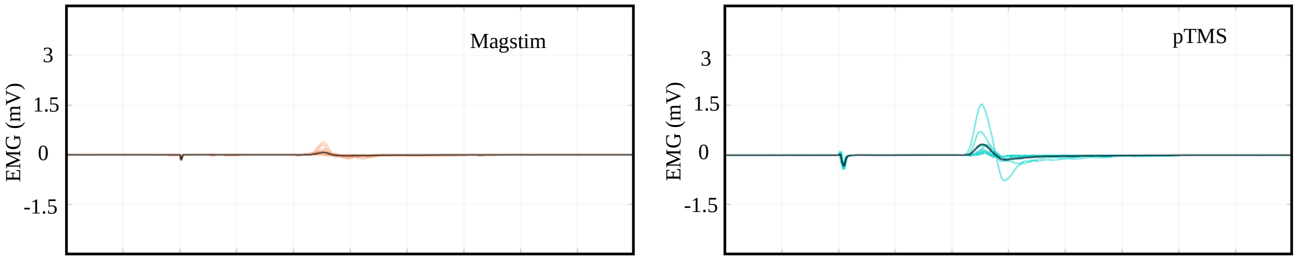

(f) (i) (ii)

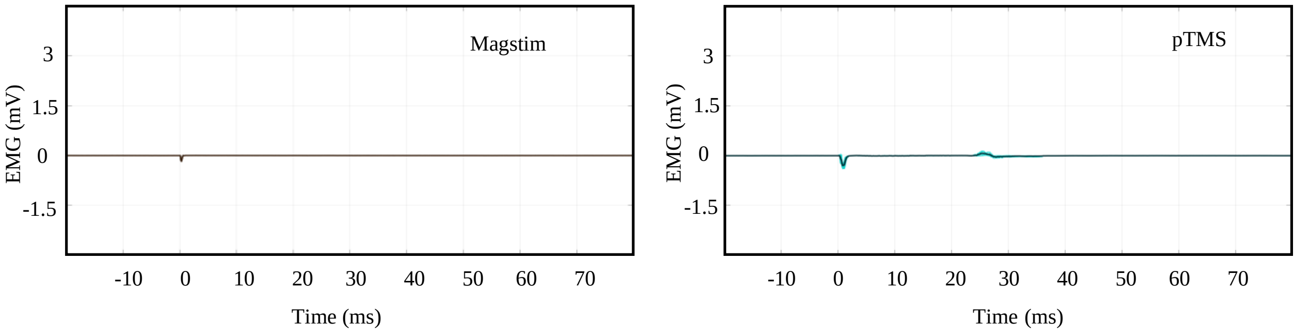

(g) (i) (ii)

Figure S3 Recorded EMG values for one subject. Stimulus intensity values are equal to (a) 50% of MSO, (b) 47% of MSO, (c) 44% of MSO, (d) 41% of MSO, (e) 38% of MSO, (f) 35% of MSO and (g) 32% of MSO of the Magstim 200. For each intensity, (i) shows the EMG response to the Magstim 200 stimuli, (ii) shows the EMG response to the pTMS2 stimuli. The average EMG is shown by a black line.

Table S1 Participant characteristics

ID	Gender	RMT for Magstim	RMT for pTMS
1	F	43	40
2	F	33	32
3	M	40	38
4	F	40	35
5	F	48	44
6	M	47	43
7	F	44	41
8	F	35	32
9	F	42	38
10	F	36	32
11	M	53	50
12	M	35	31

Individual input-output curves

The least-squares curve regression was utilized to extract the parameters for the IO curves. Curves were fitted in a mixed Gaussian-type model in the log-transformed domain:

$$y = y_{low} + (y_{low} - y_{high}). \Phi\left(\frac{V_{pulse} - V_{midpoint}}{S}\right) \qquad (1)$$

Where y is the log-transformed peak-to-peak response amplitude, V_{pulse} is the TMS pulse amplitude as a percentage of the maximum stimulator output, $V_{midpoint}$ is the midpoint of the slope, y_{low} is the lower saturation level, y_{high} the is upper saturation level, S is the stretch and Φ is the cumulative Gaussian function [23]. In general, the MEP responses have a non-Gaussian distribution, but we have not tested for normality here as the sample size is small which often makes those tests unreliable [24]. We transformed the data into the log domain to make the distributions more normal [25].

The slope of the IO curves was calculated from the tangent at the point where 50% of the maximum MEP size was reached. For two of the participants, who had a high threshold, we could not reach a plateau value for the IO curve, therefore these curves were excluded from the slope comparison.

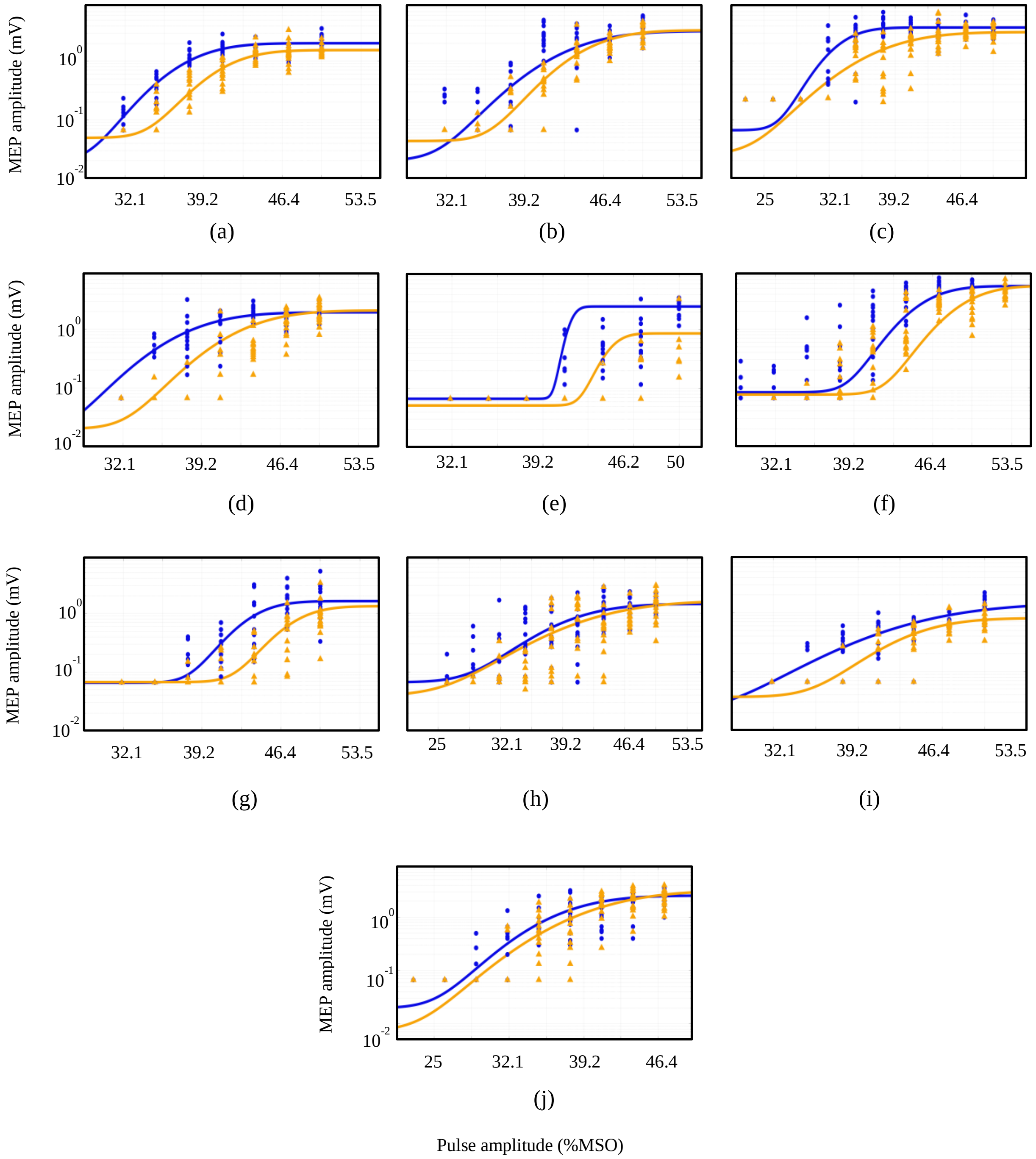

Figure S4 Individual input-output curves for the participants for both devices. Each dot represents an individual data point, and the solid lines show the best line of fit. The vertical axis is on a logarithmic scale. The blue lines and dots are related to the pTMS device and the orange lines and dots are related to the Magstim device.

Measurement of applied pTMS2 pulse

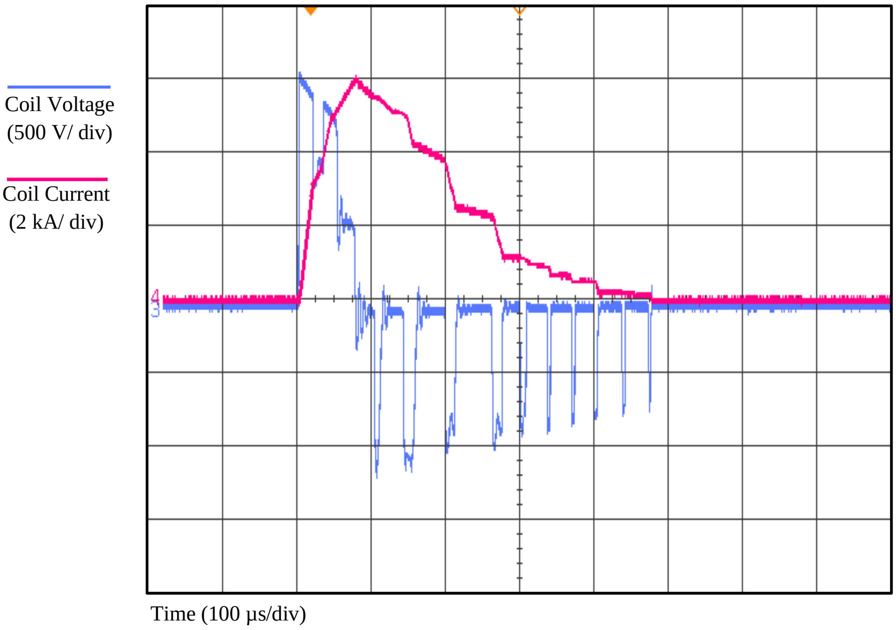

Figure S5 The measured waveform for the PWM-equivalent of the monophasic stimulus. The coil voltage and current were measured via a high-voltage differential probe (TA044, PICO TECHNOLOGY, UK) and a Rogowski current probe (I6000S FLEX-24, FLUKE, USA), respectively. A digital oscilloscope with a sampling rate of 500 Ms/s was utilized in all measurements. No bandwidth restrictions or filters have been embedded to remove switching spikes.

References

[1] N. Gattinger, G. Moßnang and B. Gleich, "flexTMS-a novel repetitive transcranial magnetic stimulation device with freely programmable stimulus currents.," *IEEE Trans Biomed Eng,* vol. 59, no. 7, pp. 1962-70, Jul 2012.

[2] A. V. Peterchev, K. D'Ostilio, J. C. Rothwell and D. L. Murphy, "Controllable pulse parameter transcranial magnetic stimulator with enhanced circuit topology and pulse shaping.," *Journal of neural engineering,* vol. 22, no. 5, 2014.

[3] M. Memarian Sorkhabi and et al., "Programmable Transcranial Magnetic Stimulation- A Modulation Approach for the Generation of Controllable Magnetic Stimuli," *IEEE Transactions on Biomedical Engineering,* vol. 68, no. 6, pp. 1847 - 1858, 2020.

[4] J. O. Nieminen and et al., "Multi-locus transcranial magnetic stimulation system for electronically targeted brain stimulation," *Brain stimulaiton,* vol. 15, no. 1, pp. 116-124, 2022.

[5] L. M. Koponen and et al., "Multi-locus transcranial magnetic stimulation—theory and implementation," *Brain stimulaiton,* vol. 11, no. 4, pp. 849-855, 2018.

[6] L. M. Koponen and et al., "Noninvasive extraction of microsecond-scale dynamics from human motor cortex," *Human Brain Mapping,* vol. 39, no. 6, pp. 2405-2411, 2018.

[7] A. E. Tervo and et al., "Closed-loop optimization of transcranial magnetic stimulation with electroencephalography feedback," *Brain stimulation,* vol. 15, no. 2, pp. 523-531, 2022.

[8] Z. Zeng and et al., "Modular multilevel TMS device with wide output range and ultrabrief pulse capability for sound reduction," *Journal of Neural Engineering,* vol. 19, no. 2, 2022.

[9] B. Lohse and et al., "The Modular Multilevel Magnetic Stimulator: Energy-Efficiency, Pre-Charging and Overlap Protection," in *IECON 2021 – 47th Annual Conference of the IEEE Industrial Electronics Society*, Toronto, 2021.

[10] S. M. Goetz and et al., "Circuit topology and control principle for a first magnetic stimulator with fully controllable waveform," in *Annual International Conference of the IEEE Engineering in Medicine and Biology Society*, San Diego, 2012 .

[11] Y. P. Siwakoti and et al., "Chapter 1 - Power Electronics Converters—An Overview," in *Control of Power Electronic Converters and Systems*, Academic Press, 2018, pp. 3-29.

[12] M. Memarian Sorkhabi and T. J. Denison, "A neurostimulator system for real, sham, and multi-target transcranial magnetic stimulation," *Journal of Neural Engineering,* vol. 19, 2022.

[13] A. Marquez and et al., "Variable-Angle Phase-Shifted PWM for Multilevel Three-Cell Cascaded H-Bridge Converters," *IEEE Transactions on Industrial Electronics,* vol. 64, no. 5, pp. 3619 - 3628, 2017.

[14] E. Barbie and et al., "Closed-Form Analytic Expression of Total Harmonic Distortion in Single-Phase Multilevel Inverters With Staircase Modulation," *IEEE Transactions on Industrial Electronics,* vol. 67, no. 6, pp. 5213 - 5216, 2020.

[15] J. Cao and P. Grover, "STIMULUS: Noninvasive Dynamic Patterns of Neurostimulation Using Spatio-Temporal Interference," *IEEE Transactions on Biomedical Engineering,* vol. 67, no. 3, pp. 726 - 737, 2020.

[16] M. Memarian Sorkhabi, K. Wendt and T. Denison, "Temporally Interfering TMS: Focal and Dynamic Stimulation Location," in *42nd Annual International Conference of the*

IEEE Engineering in Medicine & Biology Society (EMBC), Montreal, QC, Canada, 2020.

[17] N. Grossman and et al., "Noninvasive Deep Brain Stimulation via Temporally Interfering Electric Fields," *Cell,* vol. 169, no. 6, pp. 1029-1041, 2017.

[18] E. Mirzakhalili and et al., "Biophysics of Temporal Interference Stimulation," *Cell Systems,* vol. 11, no. 6, pp. 557-572.e5, 2020.

[19] A. S. Aberra and e. al., "Simulation of transcranial magnetic stimulation in head model with morphologically-realistic cortical neurons," *Brain stimulation,* vol. 13, no. 1, pp. 175-189, 2020.

[20] A. S. Aberra, "TMSsim_Aberra2019.," GitHub, 2019. [Online]. Available: https://doi.org/10.5281/zenodo.2488572.

[21] M. L. Hines and H. Carnevale, "The NEURON simulation environment," *Neural Comput,* vol. 15, no. 9, pp. 1179-1209, 1997.

[22] K. Wendt and et al., "Comparison between the modelled response of primary motor cortex neurons to pulse-width modulated and conventional TMS stimuli," in *43rd Annual International Conference of the IEEE Engineering in Medicine and Biology Society (EMBC),* 2021.

[23] K. D'Ostilio and et al., "Effect of coil orientation on strength–duration time constant and I-wave activation with controllable pulse parameter transcranial magnetic stimulation," *Clinical Neurophysiology,* vol. 127, no. 1, pp. 675-683, 2016.

[24] S. M.Goetz and et al., "A Novel Model Incorporating Two Variability Sources for Describing Motor Evoked Potentials," *Brain Stimulation,* vol. 7, no. 4, pp. 541-552, 2014.

[25] P. Pasqualetti and F. Ferreri, "W14.4 Amplitude values of motor evoked potentials: statistical properties and neurophysiological implications," *Clinical Neurophysiology,* vol. 122, pp. S44-S45, 2011.
